# Natural variation in CBF gene sequence, gene expression and freezing tolerance in the Versailles core collection of Arabidopsis thaliana

**DOI:** 10.1186/1471-2229-8-105

**Published:** 2008-10-15

**Authors:** Heather I McKhann, Carine Gery, Aurélie Bérard, Sylvie Lévêque, Ellen Zuther, Dirk K Hincha, S De Mita, Dominique Brunel, Evelyne Téoulé

**Affiliations:** 1 Etude de Polymorphisme des Génomes Végétaux (EPGV), INRA, CNG, 2, rue Gaston Crémieux, 91057 EVRY Cedex, France; 2 Station de Génétique et Amélioration des Plantes (SGAP), INRA, Route de Saint Cyr, 78026 Versailles Cedex, France; 3 Max-Planck-Institute of Molecular Plant Physiology, Am Mühlenberg 1, D-14476 Potsdam, Germany; 4 INRA, UMR 1097 "Diversité et Adaptation des Plantes Cultivées", Domaine de Melgueil, 34130 Mauguio, France; 5 UPMC, 4 place Jussieu, 75252 Paris Cedex 05, France

## Abstract

**Background:**

Plants from temperate regions are able to withstand freezing temperatures due to a process known as cold acclimation, which is a prior exposure to low, but non-freezing temperatures. During acclimation, a large number of genes are induced, bringing about biochemical changes in the plant, thought to be responsible for the subsequent increase in freezing tolerance. Key regulatory proteins in this process are the CBF1, 2 and 3 transcription factors which control the expression of a set of target genes referred to as the "CBF regulon".

**Results:**

To assess the role of the CBF genes in cold acclimation and freezing tolerance of Arabidopsis thaliana, the CBF genes and their promoters were sequenced in the Versailles core collection, a set of 48 accessions that maximizes the naturally-occurring genetic diversity, as well as in the commonly used accessions Col-0 and WS. Extensive polymorphism was found in all three genes. Freezing tolerance was measured in all accessions to assess the variability in acclimated freezing tolerance. The effect of sequence polymorphism was investigated by evaluating the kinetics of CBF gene expression, as well as that of a subset of the target COR genes, in a set of eight accessions with contrasting freezing tolerance. Our data indicate that CBF genes as well as the selected COR genes are cold induced in all accessions, irrespective of their freezing tolerance. Although we observed different levels of expression in different accessions, CBF or COR gene expression was not closely correlated with freezing tolerance.

**Conclusion:**

Our results indicate that the Versailles core collection contains significant natural variation with respect to freezing tolerance, polymorphism in the CBF genes and CBF and COR gene expression. Although there tends to be more CBF and COR gene expression in tolerant accessions, there are exceptions, reinforcing the idea that a complex network of genes is involved in freezing tolerance and that the CBF genes alone cannot explain all differences in phenotype. Our study also highlights the difficulty in assessing the function of single transcription factors that are members of closely related gene families.

## Background

The ability of plants to survive freezing temperatures depends on their capacity to cold acclimate, that is, to prepare for freezing temperatures during exposition to low, but non-freezing, temperatures [1]. During this period of cold acclimation, a number of biochemical and physiological changes takes place that include modification of the lipid composition of membranes and an increase in total soluble protein and other molecules, such as sugars and proline, thought to serve as cryoprotectants (see [2] for review). As a consequence, plants from temperate regions can survive freezing at temperatures ranging from -5°C to -30°C, depending on the species. Non-acclimated wheat, for instance, is killed at freezing temperatures around -5°C but following cold acclimation is able to survive temperatures down to -20°C [1]. In contrast, most plants from tropical regions, for example tomato and maize, are unable to acclimate and to survive freezing.

Cold acclimation is associated with large modifications of gene expression [3-6]. In Arabidopsis, a central pathway includes the CBF/DREB1 (CRT/DRE binding factor/DRE-binding factor 1) genes. It is estimated that 12–20% of cold-induced transcriptional changes are accounted for by CBF1-3 [7]. These AP2/ERF type transcription factors are rapidly induced in response to cold and reach a peak of expression after 2 to 3 h of exposure to cold [8-10]. The CBF proteins in turn activate expression of a set of target effector genes by binding to a core sequence in their promoter, alternatively called the C-repeat (CRT), the dehydration-responsive element (DRE) or the low temperature response element (LTRE), that is involved in cold responsiveness [11,12]. Genes containing this motif are known as COR (cold regulated), (E)RD ((early) responsive to dehydration), KIN (cold induced) or LTI (low temperature-induced) genes, and are collectively referred to as the "CBF regulon".

CBF genes appear to be ubiquitous in plant species and are almost always present as a gene family (e.g. [13,14]). In Arabidopsis, there are four characterized CBF genes: CBF1, 2 and 3, located in tandem on chromosome 4, are cold induced, while CBF4 is reported to be involved in drought tolerance [15]. Homologues have been described in many species including wheat, rye, and Brassica napus, all of which can acclimate, and even in tomato, which is chilling-sensitive [16,17]. Some species have large CBF gene families, for example barley, which has at least 20 family members [13]. CBF homologues are also present in tree species including poplar [18] and Eucalyptus [14]. Not all these homologous genes are cold inducible. Tomato, for example, has three CBF genes but just one is cold inducible; moreover, tomato exhibits a reduced CBF regulon, which may contribute to its freezing sensitivity [16].

In addition to their ubiquitous presence in plants, major QTL for freezing tolerance have been identified in both Arabidopsis [19] and wheat [20] that localize to the region of the CBF genes. Moreover, it has been found that over-expression of any individual Arabidopsis CBF gene leads to constitutive expression of CBF regulon genes and an increase in freezing tolerance without cold exposure [10,21-23]. The CBF genes thus seem to play a critical role in cold acclimation leading to freezing tolerance. It is unclear, however, to what extent the three Arabidopsis CBF genes have redundant roles. The same target genes are induced following over-expression of each CBF, leading to the conclusion that the CBF genes are functionally redundant [23]. On the other hand, there is evidence that CBF2 has a regulatory role. In the only CBF mutant described to date [24], there is a T-DNA insertion in the putative TATA box upstream of the CBF2 gene which leads to plants that are more freezing tolerant in the non-acclimated and the acclimated state, indicating that CBF2 is a negative regulator of freezing tolerance. This is supported by RNAi and anti-sense lines that indicate that CBF2 differs in function from CBF1 and 3 [25]. The lack of characterized mutants has so far precluded analysis of loss-of-function phenotypes for the CBF1 and CBF3 genes.

The study of natural genetic variation has proven to be an alternative means to elucidate the functional role of candidate genes in a given process (see [26]). Previously, a core collection of 48 Arabidopsis thaliana accessions was generated that maximizes genetic variation in this species [27]. Further, it is known that Arabidopsis plants of different geographic provenance differ in their ability to cold acclimate [19,28-30]. Thus, it could be expected that naturally-occurring phenotypic variation in freezing tolerance could be related to polymorphisms present in the CBF genes and their promoter regions, and that in particular the inability to withstand freezing might be traced back to deleterious polymorphisms in these genes. We therefore chose to characterize the polymorphism present in the CBF1, 2 and 3 genes and their promoters in the Versailles Arabidopsis core collection. Polymorphism in the CBF genes could exert an effect at two different levels: in the expression of the CBF genes themselves, via polymorphism in the respective promoters, or in the expression of the downstream effector genes (COR genes) via polymorphism in the CBF coding regions. A set of eight accessions was chosen to perform a detailed analysis of individual CBF and COR gene expression during acclimation, using quantitative PCR (Q-PCR). Finally, freezing tolerance phenotype was determined in the entire core collection and the LT_50 _was determined from electrolyte leakage measurements in the eight accessions to better understand the relation between CBF gene polymorphism, CBF and COR gene expression and acclimated freezing tolerance.

## Results

### Sequencing of the CBF genes in the Arabidopsis core collection

We surveyed the sequence diversity of the CBF genes to understand their role in gene expression variation, but also to determine if these genes exhibit atypical patterns of polymorphism that are suggestive of recent selection. The entire coding regions of the CBF1, 2 and 3 genes as well as 1200–1400 bp upstream of the ATG were sequenced in the 48 accessions of the Versailles core collection. We additionally sequenced the accessions WS and Col-0, used as genetic background for many mutant lines and for over-expression lines of CBF genes [4,22,23]. Table 1 summarizes the sequencing results. All three genes were found to be highly polymorphic, particularly in their promoters, with CBF1 the most and CBF2 the least polymorphic gene. This ranking was consistent between promoters and coding regions. In the coding regions, a number of non-synonymous amino acid changes were found, as described in detail below.

**Table 1:** Summary of sequencing results

		**CBF1**	**CFB2**	**CFB3**	
	**no. accessions sequenced**	**48**	**48**	**48**	
**promoter**	bp	1210	1430	1314	**CBF1**: 4 deletions: 8–50 bp
	nb SNP	106	65	81	3 insertions: 9–20 bp, 1 large insertion
	SNP/100 bp	8,8	4,5	6,2	**CBF2**: deletions 50 bp, 1.5 kb
					2 insertions, 1 microsatellite
	**no. accessions sequenced**	**48**	**48**	**46**	**CBF3**: insertion 9 bp

**coding sequence**	bp	642	652	651	**CBF1**: deletion 10 AA
	nb AA	213	216	216	**CBF3**: 1 codon stop
	nb SNP	39	20	27	
	SNP/100 bp	5,1	3,1	4,1	
	SNP ns	12	8	14	

"SNP ns" refers to the number of non-synonymous amino acid changes

Additional file 1 summarizes the polymorphism data for the three genes. Tajima's D statistic was used to evaluate the allele frequency spectrum and quantify the excess of rare alleles (rare alleles generate more negative D values). Two observations could be suggestive of a recent selective sweep in CBF2: a lower amount of polymorphism relative to the other genes and more negative and significantly lower values of Tajima's D (indicating an excess of rare, therefore recent, alleles). However, (1) a large scale survey of nucleotide sequence variation in A. thaliana shows that the average distribution of Tajima's D in the genome is biased towards negative values [31], (2) sequencing of 10 fragments to generate the core collection also yielded negative Tajima's D [27]. Therefore, negative values of D cannot be explained unambiguously by selection rather than by demographic processes (e.g. demographic growth). Last, levels of selective constraints on the segregating amino acid polymorphism, as measured by the ratio π_ns_/π_S_, was fairly constant across the three genes (0.11–0.21) despite a tenfold variation in π. This ratio was highest in the coding region of CBF2 but only slightly higher than in the coding region of CBF3.

### CBF gene promoter region polymorphism

In the promoter regions, a number of insertions and deletions were found, and the accession Cvi-0 (166 AV) was distinguished by a deletion of 1.6 kb in the CBF2 promoter ending at position -295 (where 1 is the A of ATG; position 1136 in our numbering). An (AT) microsatellite was found in the promoter of CBF2 (-676 to -640; positions 755–791 in our numbering). Although few functional studies of the CBF gene promoters have been undertaken, one region defining the motifs ICEr1 and ICEr2 has been shown to be critical to CBF2 gene expression, while several MYC sites are located in the CBF3 promoter and one, located at -179 to -160 (MYC2, [32]; 289–308 in our numbering), seems critical for ICE1 binding to the CBF3 promoter [32,33]. A number of conserved sequences between the CBF1, 2 and 3 gene promoters have also been identified and are known as boxes I-VI [34]. Further, a number of MYB recognition sequences in the promoters are postulated to play a role in the binding of MYB15 to the CBF promoters [35]. All these regions are postulated to be important for the (co-) regulation of the three CBF genes.

We also examined individual CBF promoters using the database of orthologous promoters (DoOP, [36]) which searches for common motifs in orthologous promoters. A number of motifs were identified in each of the three genes, as shown in Additional file 2. These motifs, conserved among several species, are thought to play a regulatory role. As seen in Additional file 2, some of these motifs lie within the six "CBF boxes" cited above.

### Freezing tolerance phenotype of the investigated accessions

**Figure 1:**
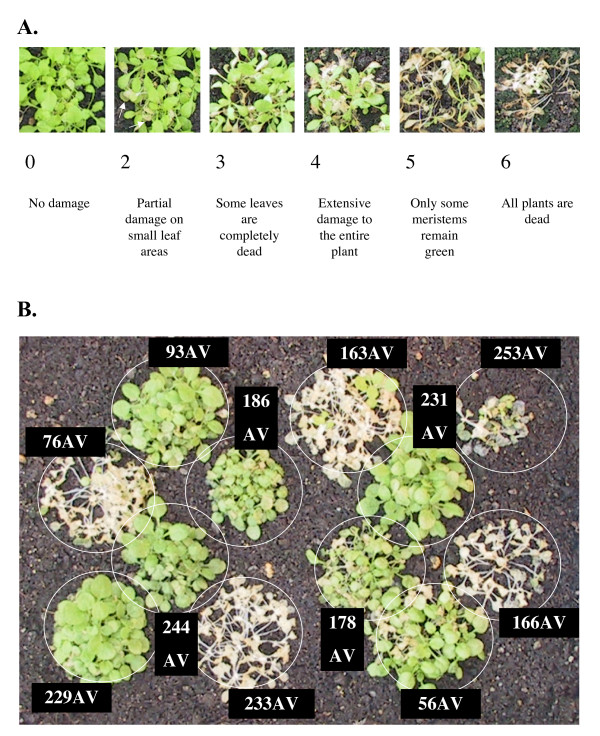
**Phenotyping the core collection following acclimation and a freezing period of -5°C for 48 hours**. A. Scale of notation from 0 (no damage) to 6 (dead plants). B. Example of a flat containing accessions with contrasting phenotypes. Each accession was noted at least 3 times and a mean score was calculated. Plants were photographed after 5 days of recovery.

Freezing tolerance was evaluated in the entire Arabidopsis thaliana Versailles core collection of 48 accessions, as well as WS (244 AV) and Col-0 (186 AV), by acclimating plants for 7 days at 5°C followed by 48 h freezing at -5°C. Recovery of the plants was noted at 5 and 8 days after thawing (Fig. 1, Table 2). As expected [29], Cvi-0 (166 AV) and Can-0 (163 AV) were among the most sensitive accessions, while WS (244 AV) and Rld-2 (229 AV) showed the expected high degree of freezing tolerance [21]. Col-0 (186 AV), on the other hand, showed unexpectedly extensive damage under these conditions (compare e.g. [37]).

**Table 2:** Phenotyping of the Versailles core collection.

**N°**	**Name**	**Origin**	**Mean at day 5**	**Rank at day 5**	**Mean at day 8**	**Rank at day 8**	**LT_50_**
70 AV	Kn-0	Lituania	0.0	1	0.7	1	
215 AV	Mh-1	Poland	1.0	4	1.0	2	
**229 AV**	**Rld-2**	**Russia**	**1.0**	**4**	**1.0**	**2**	**-9.1**
**231 AV**	**Rub-1**	**Ukraine**	**1.3**	**5**	**1.0**	**2**	**-10.7**
**244 AV**	**WS**		**1.0**	**4**	**1.0**	**2**	**-10.6**
236 AV	Shakdara	Tadjikistan	0.7	2	1.3	3	
92 AV	Stw-0	Russia	1.3	6	1.7	4	
91 AV	Tsu-0	Japan	0.8	3	1.9	5	
93 AV	Ms-0	Russia	2.2	11	2.0	6	
40 AV	Pi-0	Austria	2.3	12	2.0	6	
162 AV	Ct-1	Italy	1.6	8	2.1	7	
160 AV	Ri-0	Canada	2.3	13	2.6	8	
94 AV	Mt-0	Libya	2.5	15	2.7	9	
235 AV	Sav-0	Czech Rep.	2.7	16	2.7	9	
63 AV	Lip-0	Poland	1.8	9	2.7	9	
224 AV	Oy-0	Norway	2.3	12	3.0	10	
53 AV	Sp-0	Germany	2.7	16	3.0	10	
206 AV	Jm-0	Czech Rep.	2.5	14	3.3	11	
172 AV	Bur-0	Eire	3.2	18	3.3	12	
197 AV	Enkheim-T	Germany	3.6	19	3.3	12	
266 AV	N13	Russia	2.3	13	3.5	13	
263 AV	N7	Russia	4.5	27	3.7	14	
42 AV	Bl-1	Italy	2.7	16	3.9	15	
252 AV	Akita	Japan	3.6	20	4.2	17	
**186 AV**	**Col-0**		**4.3**	**26**	**4.5**	**18**	**-9.7**
200 AV	Gre-0	USA	5.0	32	4.6	19	
56 AV	Ta-0	Czech Rep.	2.7	16	4.7	20	
250 AV	Yo-0	USA	3.8	21	4.7	20	
178 AV	Alc-0	Spain	4.0	23	4.8	21	
180 AV	Blh-1	Czech Rep.	4.5	27	5.0	22	
8 AV	Dijon-8	France	4.5	27	5.0	22	
234 AV	Sap-0	Czech Rep.	4.6	29	5.0	22	
257 AV	Sakata	Japan	4.5	28	5.3	23	
262 AV	N6	Russia	4.8	30	5.3	23	
50 AV	Pa-1	Italy	4.3	25	5.3	23	
190 AV	Condara	Tadjikistan	4.9	31	5.7	24	
95 AV	Nok-1	Netherlands	4.0	22	5.7	24	
*157 AV*	*Ita-0*	*Morocco*	*5.8*	*34*	*5.8*	*25*	*-6.0*
267 AV	N14	Russia	5.3	33	5.9	26	
*166 AV*	*Cvi-0*	*Cape Verde Island*	*6.0*	*35*	*6.0*	*27*	*-7.5*
25 AV	Dijon-25	France	5.8	34	6.0	27	
101 AV	Ge-0	Switzerland	4.0	23	6.0	27	
*163 AV*	*Can-0*	*Canary Islands*	*6.0*	*35*	*6.0*	*27*	*-5.8*
62 AV	St-0	Sweden	4.0	23	6.0	27	
83 AV	Edi-0	United Kingdom	4.5	28	6.0	27	
*233 AV*	*Sah-0*	*Spain*	*6.0*	*35*	*6.0*	*27*	*-5.4*
253 AV	Ishikawa	Japan	6.0	35	6.0	27	
76 AV	Bla-1	Spain	5.8	34	6.0	27	
68 AV	Te-0	Finland	4.1	24	6.0	27	
21 AV	Dijon-21	France	6.0	35	6.0	27	

Accessions were grown as described in the methods, acclimated for 7 days at 5°C, and then exposed to -5°C for 48 hours. Notes were given as described after 5 days and 8 days of recovery. Accessions are sorted from freezing tolerant to freezing sensitive at day 8 (top to bottom). The accessions Col-0 and WS, which are not in the core collection, were also included. Freezing tolerant cold core accessions are in bold and freezing sensitive in italics For LT50 measurements, see Methods.

For comparison with the whole plant survival assay, eight accessions were additionally used for electrolyte leakage measurements. Among the sensitive accessions, we chose Cvi-0 (166 AV), Ita-0 (157 AV), Can-0 (163 AV) and Sah-0 (233 AV), among the intermediate ones, Col-0 (186 AV), and among the tolerant accessions, WS (244 AV), Rld-2 (229 AV), and Rub-1 (231 AV). In these experiments, 42 day old plants were acclimated for 14 days at 4°C. Detached mature leaves were frozen and thawed under highly controlled conditions and LT_50 _values (temperature of 50% electrolyte leakage) were calculated (compare [28]). The results (Table 2), were consistent with the whole plant survival assay, except for Col-0 (186 AV), which showed greater freezing tolerance in the electrolyte leakage assay. So finally the eight accessions fall into two contrasting groups with respect to their freezing tolerance, a tolerant and a sensitive one (Table 2). For brevity, we call these eight accessions the "cold core" in the remainder of the paper.

### Cold induced CBF gene expression

As a direct evaluation of the effect of polymorphism in the CBF promoters, we examined expression of the CBF genes in response to low temperature. Since it was not possible to study expression in all accessions, we chose the above described "cold core" for detailed analysis. Sequencing revealed that the eight accessions of the cold core differ in genotype (our results).

For this cold core, we first performed a detailed analysis of CBF promoter regions thought to be functionally important (Additional files 2 and 3). For CBF1, two polymorphisms are present in the conserved boxes, which are also present in a number of accessions outside the cold core. On the other hand, the sensitive accessions Sah-0 (233 AV) and Ita-0 (157 AV) contain a polymorphic region between boxes II and III that includes a stretch of 16–17 "A" that prevent sequencing in the 3'-> 5' direction. In the 5'-3' orientation, a sequence was found that showed no significant homology to sequences from Arabidopsis or any other species by BLAST searches. Analysis by gel electrophoresis indicated that these promoters have an insertion that is estimated to be around 200 bp long. For the CBF2 promoter, no polymorphism was found in the conserved boxes, except for Cvi-0 (166 AV), which has a deletion covering boxes I-IV and thus the ICEr1 region. In CBF3, four polymorphisms were detected in the region of the conserved boxes, with one, (G->A at position 1002, corresponding to box IV and the ICEr1 region) only present in the sensitive accession Sah-0 (233 AV). In the MYC sites, three polymorphisms were identified. The tolerant accession Rub-1 (231 AV) had polymorphisms in the MYC2 and MYC5 sites, which were shared with three other accessions. An additional polymorphism was found in the MYC5 site that was present in three of the sensitive accessions as well as in four other accessions. In the Myb recognition sequences that showed medium or strong binding to MYB15 [35], the only significant polymorphism is present in Cvi-0 (166 AV), which lacks all four recognition sequences (not shown). In the motifs identified by DoOP, there was little polymorphism even among all 50 accessions (Additional file 2). Among the cold core, one polymorphism was found in motif 8 of the CBF3 promoter in Ita-0 (157 AV).

We also examined the occurrence of rare SNPs in the three CBF promoters in the cold core. Of 16 singletons (present only once in all 50 accessions) in the CBF1 promoter, seven were found in the four sensitive accessions. In CBF2, 10 out of 29 singletons were in these accessions and in CBF3, 17 out of 36. No singletons were present in the four freezing tolerant accessions of the cold core.

**Figure 2:**
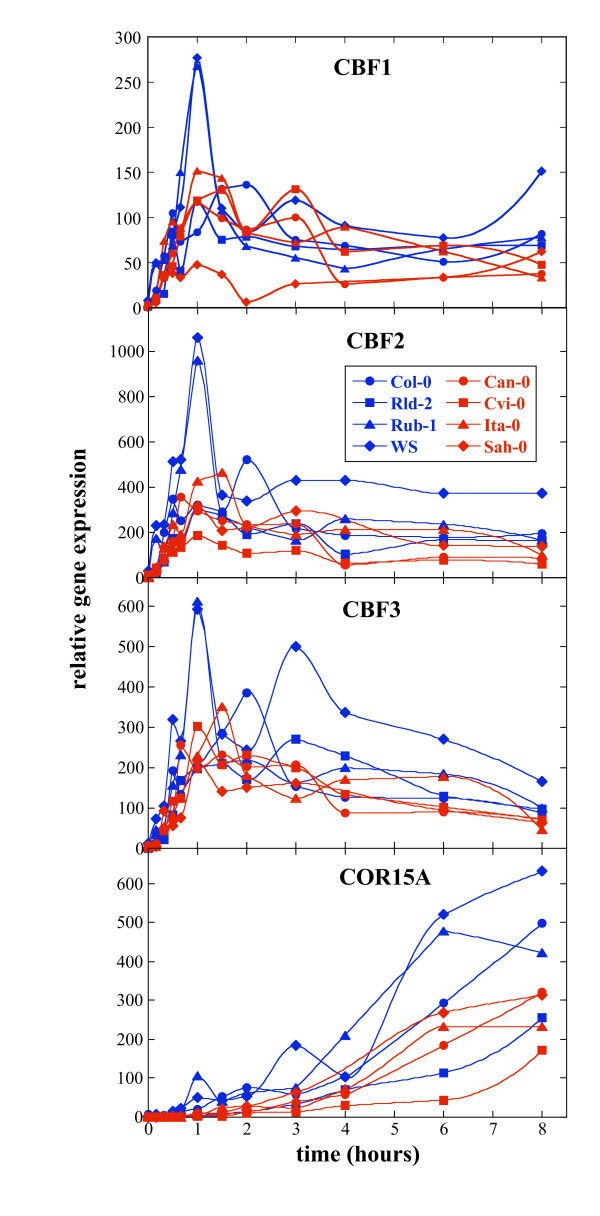
**Expression of the CBF genes and COR15a over 8 hours in the cold**. Kinetics of induction of CBF1, 2 and 3 and COR15a by cold. RNA was prepared by protocol 1 (see Methods) and PCR was performed on the ABI 7900 HT platform. For each gene, eight accessions were analyzed by Q-PCR: 157 AV (Ita-0), 163 AV (Can-0), 166 AV (Cvi-0), 186 AV (Col-0), 229 AV (Rld-2), 231 AV (Rub-1), 233 AV (Sah-0), and 244 AV (WS). Sensitive accessions are in red and tolerant ones in blue. Time points shown are 10, 20, 30, 40 min, 1 h, 1.5 h, 2 h, 3 h, 4 h, 6 h, 8 h after transfer to 5°C. Average standard errors in the determination of the expression of the different genes varied between 0.16 Ct and 0.36 Ct.

**Figure 3:**
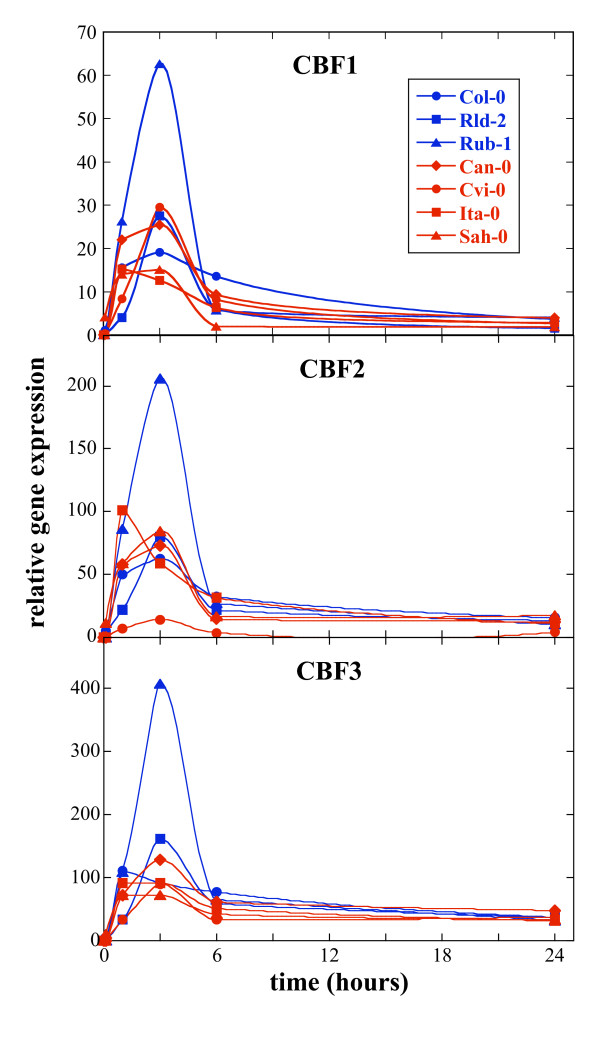
**Expression of the CBF genes over 24 hours in the cold**. Kinetics of induction of CBF1, 2 and 3 by cold. RNA was prepared by protocol 2 (see Methods) and PCR was performed on the ABI 7900 HT platform. For each gene, seven accessions were analyzed by Q-PCR: 157 AV (Ita-0), 163 AV (Can-0), 166 AV (Cvi-0), 186 AV (Col-0), 229 AV (Rld-2), 231 AV (Rub-1), and 233 AV (Sah-0). Sensitive accessions are in red and tolerant ones in blue. Time points shown are 1 h, 3 h, 6 h and 24 h after transfer to 5°C. Average standard errors in the determination of the expression of the different genes varied between 0.38 Ct and 0.47 Ct.

We examined the kinetics of CBF expression in these eight accessions, focusing on the first 24 h of acclimation, when maximum CBF expression has been observed [8,33]. Fig. 2 provides a detailed analysis of CBF expression over the first eight hours compared to the expression of the COR gene, COR15a. Fig. 3 presents a second experiment and shows CBF expression over 24 hours. The same general trends were seen when comparing biological replicates and the different Q-PCR platforms used (data not shown; compare Methods section). All three CBF genes were expressed in all accessions, although not at the same level. A peak of CBF expression occurred between 1 and 3 h following transfer to the cold and then returned to low levels, by 24 h, which were, however, still clearly above the levels detected in nonacclimated plants (Figs. 2 and 3). In all accessions CBF1, 2 and 3 were found to be coordinately regulated (Additional file 6).

Highest CBF expression consistently occurred in the tolerant accessions Rub-1 (231 AV) and WS (244 AV). Surprisingly, the other accessions all showed quite comparable expression levels, except for Cvi-0 (166 AV), where CBF2 expression was always very low (Figs. 2 and 3). Rld-2 (229 AV), which showed high freezing tolerance (Table 2), showed variable expression of all three CBF genes. In Fig. 2, CBF expression in this accession was low, while in Fig. 3 expression was intermediate between that of Rub-1 (231 AV) and WS (244 AV) and the sensitive accessions. Expression in Col-0 (186 AV) was initially low, but showed a peak around 2 h for all three genes (Fig. 2). In the second experiment (Fig. 3), no data was collected at 2 h, thus expression appears overall to be low. The differences between the experiments in the spacing and number of time points analyzed may also explain other differences in the results, such as the maximum levels of expression observed for a given gene. In addition, it highlights the difficulty of analyzing expression kinetics since small time differences may lead to significant differences in expression level. Ita-0 (157 AV) showed expression levels similar to Col-0 (186 AV) although about half an hour earlier (at 1.5 h after transfer to 5°C). Interestingly, CBF1 expression in Sah-0 (233 AV) was much lower at the earliest time points than in Ita-0 (157 AV) (Fig. 2), although they both have an insertion in their promoters. CBF2 expression was present in Cvi-0 (166 AV) despite the large promoter deletion, albeit at very low levels. In these experiments, CBF2 was not expressed later than CBF1 and 3, in contrast to a previous report [24]. The length of the microsatellite in the promoter of CBF2 had no obvious effect on expression (data not shown).

An unexpected finding was that CBF gene expression occurred with multiple peaks (Fig. 2). This was seen in two independent experiments (not shown) and to a different extent depending on the accession. Thus, WS (244 AV) had expression peaks at 1 h and 3 h, while Col-0 (186 AV) showed a small peak at 30 min and a larger peak at 2 h. To rule out that there were differences in CBF expression between accessions at later time points, two experiments were performed in which plants were acclimated for 5 weeks and expression determined at weekly intervals. As previously reported [33] CBF expression remained low following the initial peak, and no detectable differences between accessions were observed (data not shown).

### CBF coding region polymorphism and COR gene expression

**Figure 4:**
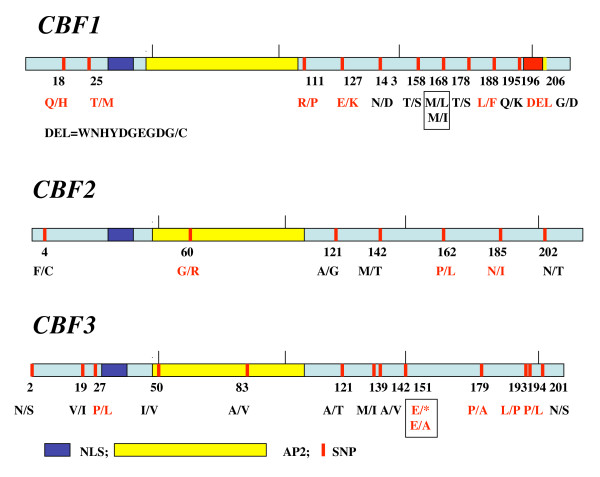
**Amino acid changes in the CBF genes of all 50 investigated accessions**. Amino acid changes in red are predicted to have an effect on protein structure.

The predicted amino acid changes in the CBF proteins of all 50 sequenced accessions are shown in Fig. 4. The majority are rare: 19/34 are singletons and an additional 10/34 are present at a frequency of less than 10% (Additional file 4). Among the non-synonymous changes, a number are predicted to have an effect on protein structure [38]. Notably, one accession has a 10 amino acid deletion at the C-terminal end of CBF1 (Bl-1; 42 AV), another a stop codon at position 151 of the CBF3 protein (Condara; 190 AV) and one (Gre-0; 200 AV) has a G->R replacement which could affect protein structure in the AP2 motif of CBF2. These are indicated in red in Fig. 4. For the eight cold core accessions, no amino acid changes were found in the NLS region or in the CBF "signature sequences" which surround the AP2 domain [17]. However, there were some amino acid changes that are predicted to alter protein structure or to affect the hydrophobic domains that are involved in trans-activation (Additional file 4). In the tolerant accessions there were only two predicted amino acid changes in CBF3, two of which are predicted to affect protein structure. They fall into a region between two hydrophobic clusters, described in [39] as important for transactivation. The first, E-> A affects Rld-2 (229 AV), but also two other accessions which are not in the cold core. The second, L->P, affects Rub-1 (231 AV) and also other accessions outside the cold core, which show intermediate tolerance.

In the four freezing sensitive accessions, the CBF proteins are more polymorphic. Of the 21 singleton polymorphisms affecting amino acids, nine are found in the four freezing sensitive accessions of the cold core. Moreover, a number of amino acid substitutions are predicted to have an effect on protein structure and are unique to sensitive accessions (CBF1, position 379, 562, 587–616; CBF2, position 554; CBF3, position 535, 581). In none of the four sensitive accessions, however, are all three CBF genes subject to polymorphism that is predicted to cause changes in protein structure.

**Figure 5:**
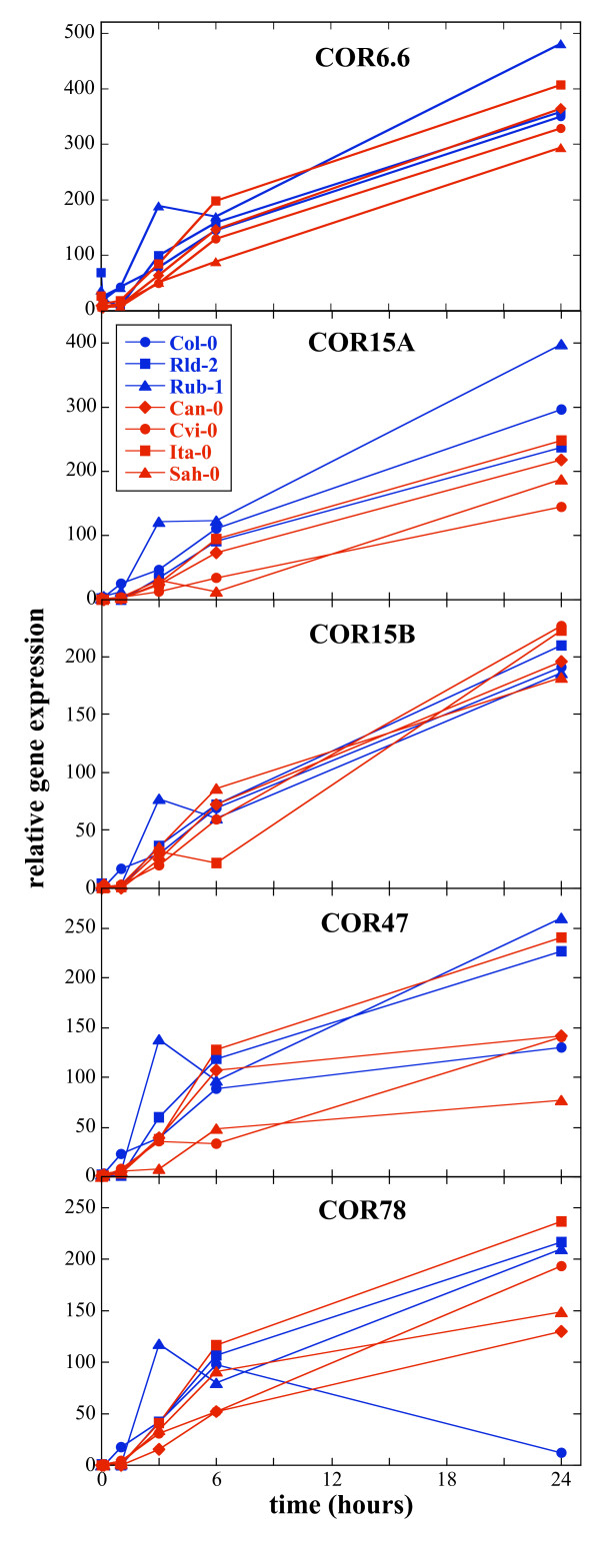
**Expression of the COR genes over 24 hours in the cold**. Expression of COR6.6, COR15a, COR15b, COR47, COR78. RNAs were the same as those used in Fig. 3. PCR was performed on the ABI7900HT platform. For each gene, seven accessions were analyzed by Q-PCR: 157 AV (Ita-0), 163 AV (Can-0), 166 AV (Cvi-0), 186 AV (Col-0), 229 AV (Rld-2), 231 AV (Rub-1), and 233 AV (Sah-0). Sensitive accessions are in red and tolerant ones in blue. Time points shown are 1 h, 3 h, 6 h and 24 h after transfer to 5°C. Average standard errors in the determination of the expression of the different genes varied between 0.15 Ct and 0.85 Ct.

In order to investigate whether the polymorphism in the coding sequences of the CBF genes has a general effect on the expression of the target COR genes, five COR genes (COR6.6, COR15a, COR15b, COR47 and COR78) were chosen for analysis (Fig. 5). The kinetics of COR gene expression differed from that of the CBF genes (compare also Additional file 6) as has been described previously [33]. Expression increased after less than 1 h in the cold and this continued up to 24 h. Cold induction was observed for all COR genes in all accessions. The freezing tolerant accession Rub-1 (231 AV) generally showed the highest level of COR gene expression. Expression of the COR genes in the sensitive accessions was often lower than in the tolerant accessions, but this was not always the case and Col-0 (186 AV) had COR gene expression levels similar to the sensitive accessions. Over a five week period, expression of COR15b stayed at a steady level but expression of COR47 decreased after 24 h in all accessions tested (data not shown).

## Discussion

QTL mapping and over-expression studies have demonstrated the importance of the CBF genes in cold acclimation. Moreover, cold-inducible CBF genes appear to be ubiquitous in plants. To further explore the mechanism by which the CBF genes enhance freezing tolerance, we analyzed the relationship between polymorphism in these genes and their expression as well as the expression of their target COR genes. Finally, the relation between gene expression and phenotypic variation in Arabidopsis accessions with different geographic origins was evaluated. The study of naturally-occurring genetic variation provides a means of analyzing gene function without inducing pleiotropic effects that are often observed when transcription factors are ectopically expressed. In the case of Arabidopsis plants constitutively overexpressing CBF genes, such pleiotropic effects were clearly evident, as the transgenic plants showed a dwarf phenotype with severely reduced growth [9,23]. We therefore undertook to characterize the Arabidopsis core collection with respect to polymorphism present in the CBF1, 2 and 3 genes and their promoters. The CBF genes were found to be highly polymorphic, both in their promoters and coding regions. In addition, we could show that there is large phenotypic variability with respect to freezing tolerance in this collection of accessions. On the basis of our phenotyping experiments, eight accessions which can be divided into a "freezing tolerant" and a "freezing sensitive" group were chosen for detailed expression studies over the first 24 h of cold acclimation. Unlike most previous studies, we used quantitative Q-PCR to provide a sensitive measure of gene expression. To assess the effects of promoter polymorphism, we used CBF gene expression as the primary phenotype, while COR gene expression was chosen as the primary phenotype for polymorphism in the CBF coding regions.

The three CBF genes were found to be expressed in all accessions, even the most freezing sensitive, despite a high degree of polymorphism in their promoters. CBF2 was expressed in Cvi-0 (166 AV), which lacks a large portion of the CBF2 promoter and which previously had been reported on the basis of northern blot results to lack CBF2 expression [19]. Using highly sensitive Q-PCR, we found that in Cvi-0, the CBF2 gene is induced by cold, albeit at low levels. It has been reported that the sequences between -189 and -65 (1090–1214 in our numbering) may be sufficient to impart cold-responsive expression [33]. Since this region is intact in Cvi-0 (166 AV), the effect of the deletion appears to be on the strength of the cold responsiveness. The CBF2 mutant in the Col-0 background described by Novillo et al. [24] completely lacks CBF2 expression whereas Cvi-0 (166 AV) retains low levels of CBF2 expression. This difference in CBF2 expression may also explain the different phenotypes between the mutant and Cvi-0, as Cvi-0 shows very low freezing tolerance, while the mutant in contrast showed increased freezing tolerance compared to wild type Col-0 plants. This has been related to the fact that CBF1 and CBF3 were expressed at higher levels in the cold in the mutant than in the wild type. Since an appropriate "wild type control" is not available for Cvi-0, the question whether the low expression of CBF2 influences the expression levels of the other CBF genes can not be answered.

Interestingly, the freezing sensitive accessions Sah-0 (233 AV) and Ita-0 (157 AV) both have insertions in their CBF1 promoters. The insertions appear to be identical, and no homology was found to other Arabidopsis sequences. Nonetheless, these accessions have different patterns of CBF1 expression (Fig. 1). The difference in expression is presumed to be unrelated to the insertions since a number of polymorphisms exist between these two accessions in the CBF1 promoter.

Next, we explored the polymorphism in the CBF genes and its effect on COR gene expression. In the coding region, a number of amino acid changes with possible functional effects were identified in the sensitive accessions. It could be possible that, like in the case of the SFR2 gene, a single mutation can give rise to a freezing sensitive phenotype [40]. To evaluate the effect of polymorphisms, we examined expression of part of the CBF regulon, which can be considered a direct phenotype of variation in CBF genes. The five COR genes contain the consensus DRE element, to which the CBF proteins have been shown to bind and are therefore direct targets of the CBF proteins. All five COR genes were expressed in all accessions, but again, sensitive accessions generally had lower COR gene expressions levels, except for Ita-0 (157 AV) (Fig. 5). Since the CBF regulon is largely coordinately regulated, this expression pattern indicates that the response is at least partially intact even in the sensitive accessions. This is in agreement with the result that CBF genes are also all expressed in every accession. In no case were potentially damaging SNPs found in all three genes in the same accession. Moreover, since there are three CBF genes, a single mutation may not have an effect, due to functional redundancy. Nonetheless, either small differences in CBF expression could contribute to an overall smaller amount of COR gene expression or a smaller portion of the CBF regulon may be expressed. It has been reported that the overall magnitude of the CBF regulon response is important in determining freezing tolerance in Arabidopsis [29] and that the CBF regulon is smaller in tomato, which is freezing sensitive [16].

In general, the most freezing tolerant accessions have the highest levels of CBF and COR gene expression and the most sensitive have lower expression levels. With the small number of accessions studied with respect to gene expression, it is difficult to confirm that there is a direct correlation between these parameters or to pinpoint the effect of specific polymorphisms on gene expression. In the future, larger gene expression studies using whole genome microarrays and a larger number of accessions would make it possible to evaluate the effect of individual SNPs and to confirm that a given haplotype corresponds to a specific expression pattern. For example, the CBF1 and CBF3 promoters of WS are in the same haplotype as accessions Pi-0 (40 AV), Mt-0 (94 AV) and Ct-1 (162 AV). We would therefore predict similar expression patterns for the two genes in these accessions. Although there are no common polymorphisms in the CBF genes and their promoters that are unique to the sensitive accessions, a number of low frequency polymorphisms that may affect CBF and/or COR gene expression are found. In the sensitive accessions, there is an accumulation of rare polymorphisms in the coding regions, giving rise to a number of amino acid changes. These independent amino acid changes may reflect a relaxation of purifying selection: cold sensitivity may not be counter-selected in regions with no or very rare freezing events. In agreement with this hypothesis, it has been shown recently that the acclimated freezing tolerance of different Arabidopsis accessions is linearly correlated with minimum habitat temperatures [29,30].

Given this correlation, we analyzed the relationship between gene expression and freezing tolerance phenotype. We chose a phenotyping protocol similar to Zhen and Ungerer [30] that assesses plant survival after freezing and not cellular damage in a specific tissue, such as leaves, to discriminate between accessions. This test also takes into account the progress during the recovery period, with some accessions maintaining a steady condition, and others tending to show more or less damage over time. This highlights the difficulty of phenotyping for freezing tolerance: depending on the time after freezing, the damage may vary. Therefore, the phenotyping we used was based on the amount of damage after eight days of recovery. As reflected by mean scores that tend to become lower, the majority of the accessions tested showed some damage following freezing, but were able to recover. This suggests that most Arabidopsis accessions can withstand mild freezing conditions (-5°C in soil) and that highly sensitive accessions (complete lethality) are rare.

In addition, the eight accessions used for detailed expression studies were also phenotyped using electrolyte leakage assays with mature leaves as another well-established method to determine freezing tolerance. With the exception of Col-0, which showed higher freezing tolerance with the electrolyte leakage than with the plant survival assay, both methods were in close agreement. The reason for the discrepancy with Col-0 has not been established yet. However, since the electrolyte leakage data reported here are in agreement with several previous studies from different laboratories (see e.g. [28,37] and references therein), we treated Col-0 as a freezing tolerant accession in all subsequent analyses.

Among the most freezing sensitive accessions, Can-0 (163 AV), Cvi-0 (166 AV), Dijon-25 (25 AV) and Sah-0 (233 AV) belong to the West Mediterranean group, defined by studying patterns of polymorphism in a set of 71 European accessions [41]. This group contains very sensitive accessions and also contains accessions with the most southern origin and consequently with the highest minimum habitat temperatures, posing the least threat of freezing.

The lack of a clear correlation between CBF and COR gene expression and freezing tolerance phenotype might have several explanations. It is known that other pathways besides the CBF pathway are involved in cold acclimation [42] and that there are complex interactions between different components involved in freezing tolerance. At this time, it is difficult to predict how these interactions may affect CBF and COR gene expression and ultimate freezing tolerance. Nonetheless, QTL mapping in a cross between Cvi and Ler [19] supports a role for the CBFs in natural variation in this response. Additional support for the role of the CBF genes comes from Hannah et al. [29] who found a correlation between CBF1 and 2 expression and freezing tolerance in different accessions after 14 days of acclimation. In this study, plants were significantly older when acclimation occurred (an average of 49 days vs. 14 days) than in our study and plants were acclimated for 14 days as opposed to 7, thus the conditions studied are not directly comparable. We and others [37,43] have already shown that the genotype as well as the length and temperature of acclimation affect freezing tolerance.

An important factor to consider when studying the CBF genes is their tandemly duplicated nature. Duplication is considered a major source for the generation of evolutionary novelty, through processes such as neo-functionalization and sub-functionalization [44,45]. Other fates for paralogous genes include the evolution of one copy into a nonfunctional pseudogene or the functional preservation of copies to increase the robustness of the genetic network [46-48]. It would appear that the latter is the case for the CBF genes. Since these genes are duplicated in many species and show nearly identical expression profiles, it seems likely that multiple copies are required to ensure an essential function. The fact that polymorphisms that might influence protein structure and function never occurred in all three CBF genes in any accession and that more polymorphisms were found in the coding regions of CBF genes from sensitive than from tolerant accessions also point in this direction. In addition, while CBF genes clearly play a role in tolerance to abiotic stress, other, as yet unknown, functions for these genes seem likely, given that they are expressed differentially in seeds, pollen, embryos and roots in Arabidopsis [49]. Thus, it is possible that, in other tissues, there may be a specialization of individual CBF gene functions.

This raises the question of the whether there is complete functional redundancy of individual CBF genes in freezing tolerance. It has been suggested that CBF2 is a negative regulator of CBF1 and CBF3 [24,25]. Our expression data did not allow us to further confirm this possibility nor to distinguish functional differences in the three genes. Instead, as previously reported, the three CBF genes showed nearly identical temporal expression patterns, regardless of their polymorphisms. Another means of exploring the functional redundancy of the CBF genes is to look at the nucleotide variation. The ratio of non-synonymous to synonymous amino acid changes gives a measure of selective constraints on the segregating amino acid polymorphism. If a gene is highly constrained by purifying selection (any amino acid change is deleterious) then one expects π_ns _<< π_s_. This ratio is fairly constant among the three genes despite marked variation in π. This indicates that all three genes are roughly equally important functionally and, furthermore, that their function confers similar constraint over the amino acid sequence. It is possible however, that sub-functionalization of these duplicates has occurred, e.g. by tissue-specific expression, induction by different signals, or activation of different COR genes, making all of them necessary to ensure maximal cold acclimation. On the other hand, this apparent redundancy complicates the search for mutants and, as seen here, their study by the exploitation of natural variation. RNAi studies targeting individual or combinations of CBF genes [25] are currently underway and will help resolve these problems.

## Conclusion

Our results indicate that the Versailles core collection contains significant variation with respect to freezing tolerance at the level of phenotype, polymorphism in the CBF genes and CBF and COR gene expression. Although there tends to be more CBF and COR gene expression in tolerant accessions, there is no simple correlation between these factors, undoubtedly due partly to the redundancy of the three genes and the number of genes and their complex interactions in the cold response gene network. The availability of the CBF sequences in 50 accessions will allow future detailed studies to relate CBF gene polymorphism to gene expression and metabolite networks and freezing tolerance and other low temperature survival phenotypes.

## Methods

### Plant material

Accessions used are from the Versailles nested core collections [27] (see Table 2 for a list). Passport data for these accessions is available at [50].

### Sequencing of the CBF genes

Genomic DNA was extracted from leaves or seedlings using a cetyltrimethylammonium bromide (CTAB) protocol in microtiter plates [51]. Sequences of the primers used for PCR and sequencing are available in Additional file 5. PCR reactions were performed as described in [52], and then the products were purified and sequenced using the BigDye Sequencing kit according to the manufacturer's instructions (ABI, Courtaboeuf, France). Sequence products were purified and loaded onto ABI3700 or ABI3730 96 capillary sequencers.

Sequence alignment and SNP detection were performed manually using Genalys software [53], available at [54]. The genomic sequence of Col-0 [55] was used as a reference. Col-0 (186 AV) was also re-sequenced. Polymorphism and sequence data is available [56].

For the analysis of nucleotide diversity, sites with 10% or more alignment gaps or missing data were excluded. We calculated two standard indices of diversity: the Watterson estimator of θ and π, the nucleotide heterozygosity. The ratio of non-synonymous to synonymous nucleotide diversity was calculated, providing an estimation of the level of functional constraint impeding amino acid variation. Tajima's D, which is a test of selective neutrality of polymorphism was computed [57] and the p value of D was estimated using 10,000 neutral coalescent simulations (using the software DnaSP version 4.10) conditioned on the number of polymorphic sites.

### Expression studies by Q-PCR

Plants were grown in the greenhouse for a period of 2 weeks and then transferred to a 5°C growth chamber as described above. Care was taken to always start cold experiments at the same time of day (10:00 am) since the CBF genes are known to show circadian expression patterns [58].

Primers were chosen for the CBF genes that were 1) gene-specific and 2) not located in regions that were polymorphic in the accessions to be studied (Table 1). COR gene primers were as described in [28]. Five different primer pairs corresponding to 4 housekeeping genes, described by [59], were tested using the GeNorm software [60] and that pair which was the most stable (GAPDH, see Table 3) was chosen for all experiments. Dissociation curves were generated for each primer pair to verify their specificity.

**Table 3:** Primers used for quantitative PCR

**Primer name**	**Sequence**	**Source**
CBF1 342U20	GTC AAC ATG CGC CAA GGA TA	this study
CBF1 513L20	TCG GCA TCC CAA ACA TTG TC	
		
CBF2 341U20	GAA TCC CGG AAT CAA CCT GT	this study
CBF2 514L20	CCC AAC ATC GCC TCT TCA TC	
		
CBF3 353U18	CAA CTT GCG CTA AGG ACA	this study
CBF3 515L18	TCT CAA ACA TCG CCT CAT	
		
COR6.6-F	GAG ACC AAC AAG AAT GCC TTC C	Rohde et al., (2004).
COR6.6-R	TGC TCT TCT CCT CAG CTT TGC	
		
COR15a-F	AAC GAG GCC ACA AAG AAA GC	Rohde et al., (2004).
COR15a-R	CAG CTT CTT TAC CCA ATG TAT CTG C	
		
COR15b-F	CAA CGA AGC CAC AAA GAA AGC	Rohde et al., (2004).
COR15b-R	CCA TCC GCC AAG GCC TCC	
		
COR47-F	ACA AGC CTA GTG TCA TCG AAA AGC	Rohde et al., (2004).
COR47-R	TCT TCA TCG CTC GAA GAG GAA G	
		
COR78-F	GCA CCA GGC GTA ACA GGT AAA C	Rohde et al., (2004).
COR78-R	AAA CAC CTT TGT CCC TGG TGG	
		
GAPDH3ÕF	TTGGTGACAACAGGTCAAGCA	Czeckowski et al., 2005
GAPDH3ÕR	AAACTTGT CGCTCAATGCAATC	

Samples for expression studies were prepared using two different protocols. In Protocol 1 (P1), 2 week old, greenhouse grown plants were transferred to a 5°C growth chamber in the same conditions as described above. Leaves were harvested into liquid nitrogen just before transfer to 5°C, and then at 10, 20, 30, 40 min, 1 h, 1.5 h, 2 h, 3 h, 4 h, 6 h, 8 h and 24 h after transfer. In Protocol 2 (P2), 3 week old greenhouse grown plants were used and were harvested at the time of transfer to 5°C and then at 1 h, 3 h, 6 h and 24 h. RNA was extracted from approximately 100 mg leaf tissue using Trizol (Invitrogen) following the manufacturer's instructions. RNA quantity was assessed using a NanoDrop (Labtech International France, Nyxor Biotech) (P1) or the Quant-iT RNA assay kit (Invitrogen) (P2), and quality was assessed by gel electrophoresis. RNA was treated with RNAse-free DNAse set (Quiagen; 30 U) using the RNeasy mini kit (Quiagen) (P1) or TURBO DNase (Ambion) (P2) as specified. Absence of genomic DNA was confirmed by PCR [59].

In P1, 500 ng of total RNA was used for first strand cDNA synthesis using RevertAid H Minus M-MuLV reverse transcriptase (Fermentas, 50 U), Ribonuclease Inhibitor (Fermentas, 40 U), oligo (dT)18 primers (1 μg) in 20 μl. Samples were diluted 5 times in water and 5 μl used for PCR. In P2, 5 μg of total RNA was used for first strand cDNA synthesis using Superscript III reverse transcriptase (Invitrogen) and oligo (dT)16 primers. Samples were diluted 8 times in water and used for PCR.

Two different PCR platforms were utilized. In one, Q-PCR was performed on the LightCycler Instrument (Roche, Meylan, France), using the LightCycler FastStart DNA Master SYBR Green I kit for PCR (Roche) according to the manufacturer's protocol. Each reaction was performed with 5 μl of 1:5 dilution of the first cDNA strands in a total volume of 20 μl.

The reactions were incubated at 95°C for 10 min to activate the hot start recombinant Taq DNA polymerase, followed by 45 cycles of 15 s at 95°C, 10 s at 55°C and 15 s at 72°C. The specificity of the PCR amplification was checked with a heat dissociation protocol (from 70 to 95°C) following the final cycle of the PCR. PCR products were then purified and sequenced.

Alternatively, Q-PCR was performed using the SYBR Green master mix (Applied Biosystems) as described by [59] on an ABI 7900 HT (Applied Biosystems). Reactions were carried out in 5 μl total volume. PCR conditions were 50°C for 2 min; 95°C for 10 min; 40 cycles of 95°C for 15 s and 60°C for 1 min. Data were analyzed using SDS 2.1 software.

In both cases, the efficiency of the primers was calculated by performing Q-PCR on several dilutions of cDNA. Efficiency of all primer pairs was between 1.87 and 2.05. The results obtained for the different genes were standardized to the constitutive GAPDH gene expression level and were expressed as Ct vs. time.

### Test of freezing tolerance

Seeds were put in 0.1% agarose at 4°C in the dark for 48 h to ensure homogenous germination. They were then sown in square pots containing organic substrate and irrigated with mineral nutrient solution once a week and with water. Plants were grown in the greenhouse for 14 days at which time they had reached the 6–8 leaf stage. Plants were then transferred to a growth chamber at 5°C under 12 h photoperiod, 70 μE m^2 ^s^1 ^light intensity and 70% relative humidity for 7 days. Acclimated plants were then exposed to freezing temperatures varying from – 4°C to – 8°C for 48 h to 96 h. At the time plants were removed from freezing conditions, they were visually inspected to verify that freezing of the plants had occurred. After this treatment, plants were put back in the greenhouse. These conditions were optimized to maximize the variation in the response. Tolerance to freezing was determined by evaluating leaf damage and capacity for continued growth at different time points during recovery. Using a method favored by agronomists [61], damage on leaves was evaluated by noting on a scale ranging from 0 (no damage) to 6 (dead plants). Plants were sown in bunches, twelve accessions per flat in a random design allowing blind notation. Each accession was repeated at least 3 times and a mean score was calculated.

In addition, electrolyte leakage assays were used to estimate freezing tolerance in a subset of accessions from the Versailles Core collection [27]. The accessions Col-0 and WS were additionally added. All plants were grown in a greenhouse on GS90 soil with fertilizer (1:1 Vermiculite) at 16 h day length with light supplementation to reach at least 250 μE m^2 ^s^1^, a temperature of 20°C and a relative humidity of 80% during the day and 18°C and 50% relative humidity during the night. After 42 days of growth, plants were transferred to a 4°C growth cabinet at 16 h day length with 90 μE m^-2 ^s^-1 ^for an additional 14 days of cold acclimation.

Freezing damage was determined as electrolyte leakage after freezing of detached leaves to different temperatures as described in detail in previous publications [28,29]. Briefly, three rosette leaves taken from three individual plants were placed in a glass tube containing 300 μl of distilled water. Tubes were transferred to a programmable cooling bath set to -1°C, a control was left on ice during the entire experiment. After 30 min of temperature equilibration at -1°C, ice crystals were added to the tubes to initiate freezing. After another 30 min, the samples were cooled at a rate of 4°C/h. Over a temperature range of -1 to -22°C, samples were taken from the bath and thawed slowly on ice. After thawing, leaves were immersed in distilled water and placed on a shaker for 16 h at 4°C. Electrolyte leakage was determined as the ratio of conductivity measured in the water before and after boiling the samples. The temperature of 50% electrolyte leakage (LT_50_) was calculated as the LOGEC_50 _value of sigmoidal curves fitted to the leakage values using the software GraphPad Prism3. Regression curves were calculated using the mean of five replicates, each of which consisted of leaves from three plants.

## Abbreviations

AV: Versailles accession number; SNP: single nucleotide polymorphism; P1, P2: protocol 1, protocol 2; Ct: threshold cycle.

## Authors' contributions

Conception and coordination of project, HIM, DB, ET; Phenotyping, CG, ET; Electrolyte leakage assay, EZ, DKH; Sequencing and sequence analysis, HIM, AB; Nucleotide diversity analysis, SDM; Expression studies, HIM, CG, SL; Data analysis, HIM, DB, ET; Drafting of manuscript, HIM, DKH. All authors read and approved the final manuscript.

## Supplementary Material

Additional file 1 **Summary of nucleotide diversity.** Overall summary of the nucleotide diversity for the 3 CBF genes and their promoters. Click here for file

Additional file 2 **Conserved motifs between orthologous promoters.** Motifs in common between orthologous CBF gene promoters and the polymorphisms present in these motifs, along with the corresponding accessions. Click here for file

Additional file 3 **Polymorphism in conserved regions of the CBF promoters.** Motifs in common in the CBF gene promoters, and the polymorphisms present in these motifs, along with the corresponding accessions. Click here for file

Additional file 6 **Coordinate expression of CBF and COR genes in the cold (PDF).** The data are replotted from Figures 3 and 5 and are organized by accession to show the coordinate expression of the three CBF genes (black lines and symbols) and three representative COR genes (green lines and symbols). Filled black circles indicate CBF1, squares CBF2 and triangles CBF3. Filled green rhombi indicate COR6.6, circles COR15A and squares COR47. Click here for file

Additional file 4 **Amino acid changes in the CBF genes.** A summary of polymorphisms giving rise to amino acid changes in the coding sequences of CBF1-3. Click here for file

Additional file 5 **Sequences of the primers used for PCR and sequencing.** Sequences of primers used for PCR and sequencing. Click here for file
