# Supplementary figures and images for: Natural variation in CBF gene sequence, gene expression and freezing tolerance in the Versailles core collection of Arabidopsis thaliana
Source: BMC Plant Biol. 2008 Oct 15;8:105. doi: 10.1186/1471-2229-8-105 (PMC2579297; doi:10.1186/1471-2229-8-105)

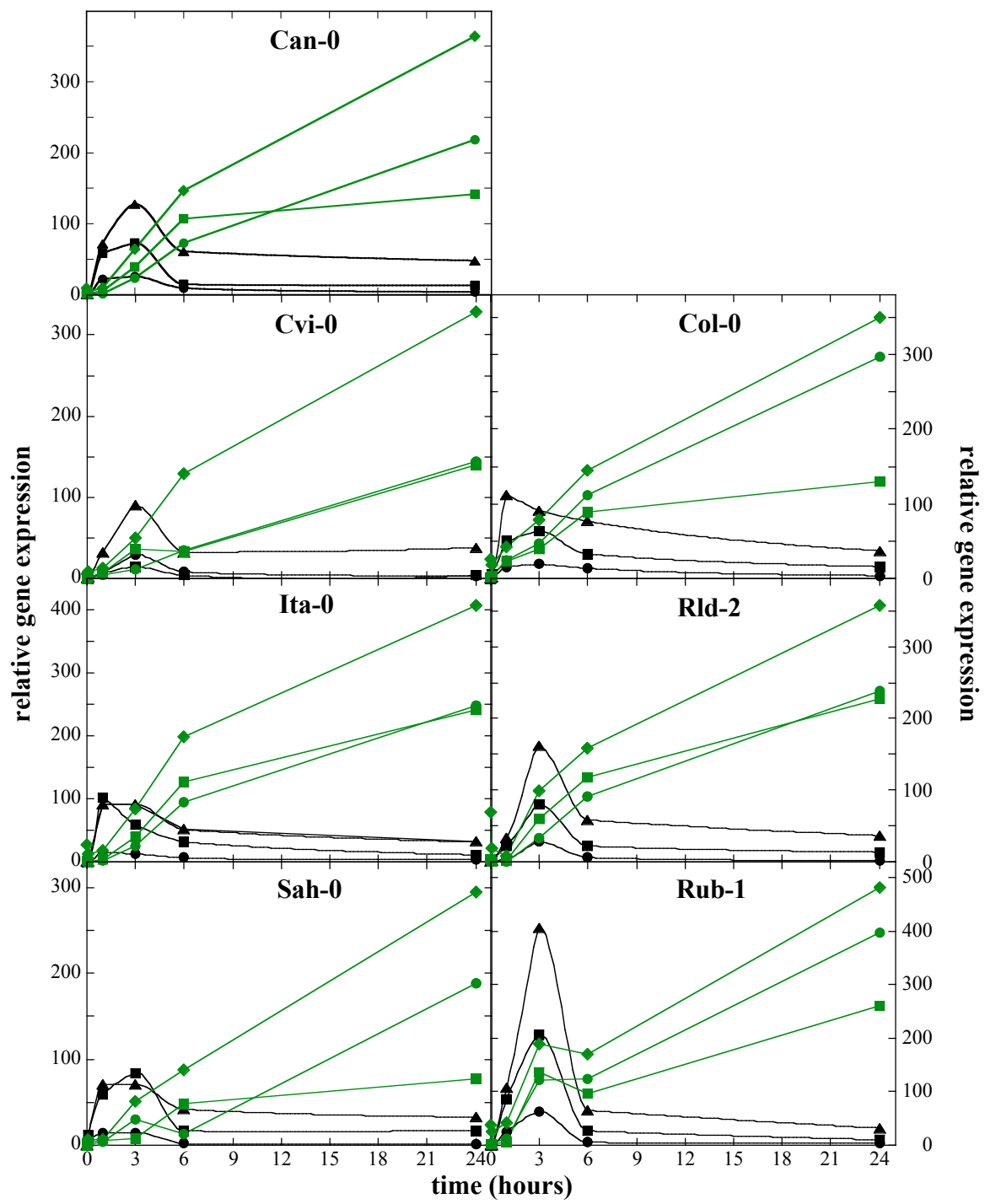

Supplement: Additional file 6 — Coordinate expression of CBF and COR genes in the cold (PDF). The data are replotted from Figures 3 and 5 and are organized by accession to show the coordinate expression of the three CBF genes (black lines and symbols) and three representative COR genes (green lines and symbols). Filled black circles indicate CBF1, squares CBF2 and triangles CBF3. Filled green rhombi indicate COR6.6, circles COR15A and squares COR47. [file 1471-2229-8-105-S6.pdf]
